# Comparative Analysis of Oncotype DX Recurrence Scores in Screen‑Detected Versus Symptomatic Early‑Stage Breast Cancer: The COSSEB Study

**DOI:** 10.7759/cureus.92141

**Published:** 2025-09-12

**Authors:** Abdalla Saad Abdalla Al-Zawi, Beata Adamczyk, Philip Idaewor, Najwa Abduljawad, Piotr Nowaczyk, Zina Aladili

**Affiliations:** 1 General and Breast Surgery, Mid and South Essex NHS Foundation Trust, Basildon, GBR; 2 General and Breast Surgery, Basildon and Thurrock University Hospital, Basildon, GBR; 3 General and Breast Surgery, Anglia Ruskin University, Chelmsford, GBR; 4 Surgical Oncology (Breast), Greater Poland Oncology Centre, Poznań, POL; 5 Histopathology/Cellular Pathology, Mid and South Essex NHS Foundation Trust, Basildon, GBR; 6 Histopathology/Cellular Pathology, Basildon and Thurrock University Hospital, Basildon, GBR; 7 Faculty of Medicine, Omar Al-Mukhtar University, Al-Bayda, Libya, Al-Bayda, LBY; 8 Surgical Oncology (Breast), Greater Poland Oncology Centre, Poznan, POL; 9 Oncology, Southend University Hospital, Southend-On-Sea, GBR

**Keywords:** breast cancer, breast conservation surgery, chemotherapy, mastectomy, oncotype dx recurrence score, screening mammogram

## Abstract

Background: Breast cancers detected through population-based screening mammography (screen-detected breast cancers, SDBCs) typically differ from cancers first identified after symptom onset (symptomatic breast cancers, SBCs) in stage and biology. We investigated whether these clinical differences extend to genomic risk as measured by the 21-gene Oncotype DX Recurrence score (ODX-RS), which guides adjuvant treatment in estrogen receptor (ER)-positive, human epidermal growth factor receptor 2 (HER2)-negative early breast cancer, and examined whether presentation mode influenced adjuvant treatment recommendations.

Methods: We performed a retrospective analysis of 200 women aged ≥50 years who underwent surgery for ER-positive, HER2-negative early breast cancer between 2017 and 2025. The cohort comprised 100 SDBCs and 100 age-matched SBCs, all of whom had low-burden axillary disease and successful ODX-RS testing. Clinicopathologic features, ODX-RS categories (≤25 vs ≥26), and adjuvant treatment recommendations were compared between groups using counts/percentages (or differences for continuous variables) with two-tailed P values; adjusted associations, where applicable, are reported as odds ratios (ORs) with 95% confidence intervals.

Results: SDBCs were smaller (median 24 mm vs 28 mm; median difference −4 mm (95% CI −7 to −0.5); P=0.05) and more frequently treated with breast-conserving surgery (77% vs 61%; P=0.01). Low HER2 expression (immunohistochemistry 1+) was more common in SDBCs (54% vs 38%; P=0.014). An ODX-RS ≥26 occurred slightly more often in SBCs than SDBCs (21% vs 17%; P=0.47), and subgroup analyses by tumor size and grade showed largely similar score distributions between groups. Adjuvant chemotherapy was recommended for 23% of SBCs and 19% of SDBC cases (P=0.49); endocrine therapy was advised for most patients in both cohorts.

Conclusions: Screen-detected and symptomatic ER-positive/HER2-negative early breast cancers exhibited broadly comparable ODX-RS distributions. Mode of presentation alone should therefore not dictate adjuvant chemotherapy decisions; multigene testing and standard clinicopathologic factors remain essential for individualized treatment planning. Larger, prospective studies are warranted to validate and extend these observations.

## Introduction

In 2022, approximately 2.3 million new cases and 670,000 deaths due to breast cancer were reported worldwide. Breast cancer is the most commonly diagnosed cancer globally and the fifth leading cause of cancer-related mortality, representing a significant global health burden, particularly among women [1,2]. Many high-income countries have implemented population-based breast cancer screening programs, which have led to reductions in both the rates of advanced cancer presentation and breast cancer-related mortality. Evidence suggests that screening women aged 50-70 years with mammography can reduce breast cancer mortality by 20%-25% [3,4].

Breast cancers detected through screening are more likely to be smaller in size, less aggressive, of lower pathological grade, and at an earlier clinical stage than tumors identified symptomatically. These tumors are also less likely to exhibit extramammary disease and are more often managed with breast-conserving surgery (BCS) [5,6]. Although screening-detected invasive breast cancers tend to be biologically indolent, their aggressiveness may increase during the preclinical phase if left undiagnosed [7].

Genomic assays play an essential role in the management of early breast cancer by aiding in risk stratification and supporting risk-adapted decisions regarding adjuvant treatment. Fewer than 10% of patients with estrogen receptor (ER)-positive, human epidermal growth factor receptor 2 (HER2)-negative early invasive breast cancer derive benefit from chemotherapy [8,9]. The Oncotype DX recurrence score (ODX-RS), a 21-gene assay, is widely used to estimate the risk of distant recurrence and the potential benefit of chemotherapy in patients with early breast cancer [10]. The validity of this assay has been established in two major studies: the TAILORx (Trial Assigning Individualized Options for Treatment) trial, involving patients with node-negative disease, and the RxPONDER (A Clinical Trial RX for Positive Node, Endocrine Responsive Breast Cancer) trial, which included patients with N1 disease (1-3 positive lymph nodes) [11-13]. This COSSEB (Comparative Analysis of Oncotype DX Recurrence Scores in Screen-Detected Versus Symptomatic Early-Stage Breast Cancer) study aims to compare ODX-RS distributions between screen-detected breast cancers (SDBCs) and symptomatic breast cancers (SBCs) and to evaluate whether presentation mode influences adjuvant treatment recommendations.

## Materials and methods

We conducted a retrospective comparative cohort study of consecutive women aged ≥50 years with ER-positive, HER2-negative early breast cancer treated within a single regional hospital network between 2017 and 2025. Patients were classified by mode of presentation as SDBC (identified through population-based screening mammography) or SBC (diagnosed after presentation with breast symptoms outside routine screening). To minimize confounding by age, SDBC and SBC cases were frequency-matched to achieve a similar age distribution at study entry.

Eligibility criteria

Inclusion criteria were ER-positive, HER2-negative invasive breast cancer; pathologic nodal status N0 or N1 with ≤3 positive nodes; definitive breast surgery; and successful 21-gene ODX-RS testing. Patients were excluded if they did not undergo ODX-RS testing or if the assay failed quality control.

Variables and outcomes

Clinicopathologic variables were abstracted, including age, tumor size, histologic subtype, grade, HER2 immunohistochemistry score, and nodal status, as well as surgical approach and adjuvant systemic therapy recommendations. The primary genomic outcome was ODX-RS, summarized in two complementary ways. First, for the primary comparative analyses, we prespecified a binary definition of high genomic risk as ODX-RS ≥26 versus ≤25. Second, for transparency and comparability with prior literature, we report the distribution across the three TAILORx-based categories: low (0-10), intermediate (11-25), and high (≥26) [11].

Justification for ODX-RS thresholds

We prespecified ODX-RS ≥26 as the clinically meaningful threshold for high genomic risk, consistent with contemporary adoption of TAILORx-derived cutoffs. The rationale for dichotomizing the ODX-RS at 26 is based on its established prognostic and predictive significance. A score of 26 or higher is associated with a substantially increased risk of breast cancer recurrence compared to lower scores. Moreover, clinical trial data, particularly from the TAILORx study [11], demonstrate that patients with ODX-RS ≥26 derive significant benefit from the addition of adjuvant chemotherapy, whereas those with lower scores typically do not. Thus, the cutoff at 26 provides a clinically meaningful threshold that helps distinguish patients likely to benefit from chemotherapy from those for whom endocrine therapy alone is sufficient [11].

Statistical analysis

Baseline characteristics were compared between SDBC and SBC using χ² or Fisher’s exact tests for categorical variables and t-tests or Mann-Whitney U tests for continuous variables, as appropriate; group comparisons are summarized with counts and percentages (or differences for continuous variables) and two-sided P-values. To evaluate whether the mode of presentation was independently associated with outcomes, we fitted multivariable logistic regression models and report adjusted odds ratios (aORs) with 95% confidence intervals. For genomic risk, the dependent variable was ODX-RS ≥26 (yes/no). For treatment recommendation, the dependent variable was adjuvant chemotherapy recommended (yes/no). In both models, the primary exposure was presentation mode (SDBC vs SBC), and we adjusted for prespecified covariates available in the dataset: age (continuous), tumor size (T1 vs T2), histologic grade (ordinal 1-3), histology (invasive lobular carcinoma vs invasive carcinoma of no special type/other), HER2 immunohistochemistry category (0, 1+, 2+), and nodal status (N0 vs N1). We assessed multicollinearity, confirmed model fit, and reported adjusted ORs with 95% CIs; two-sided P<0.05 was considered statistically significant.

Ethics

Institutional approval was obtained from the Clinical Effectiveness Unit, Mid and South Essex Hospital, UK (Approval GSURG184). The study used de-identified, routinely collected data and complied with relevant regulations and institutional policies.

## Results

**Figure 1:**
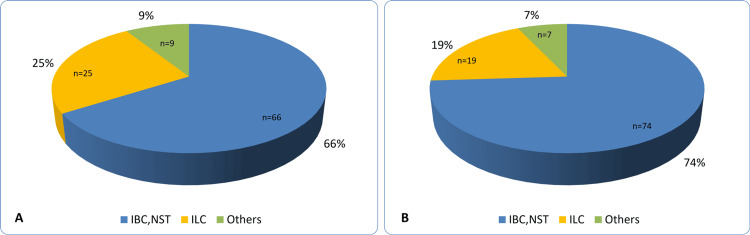
Histologic subtype distribution of (A) screen‑detected breast cancers and (B) symptomatic breast cancers IBC, NST: Invasive breast carcinoma of no special type. ILC: Invasive lobular carcinoma.

The study included 200 women treated for breast cancer between 2017 and 2025, assigned into two age-matched groups: 100 with SDBCs and 100 with SBCs. Ages ranged from 50 to 78 years, with mean ages of 62.5 ± 7.4 years in the SBC group and 62.4 ± 7.4 years in the SDBC group. Among patients with SDBCs, 12% of cases were identified at the first screening mammogram, 11% at the second, and up to 15% at the sixth or seventh screening. SBCs were more likely than SDBCs to present with larger tumors (median size 28 mm vs 24 mm; median difference -4 mm (95% CI, -7 to -0.5); P=0.5) and more frequently with invasive carcinoma of no special type (74% vs 66%; OR=1.46; 95% CI, 0.01 to 0.2; P=0.21; Figure 1).

**Figure 2:**
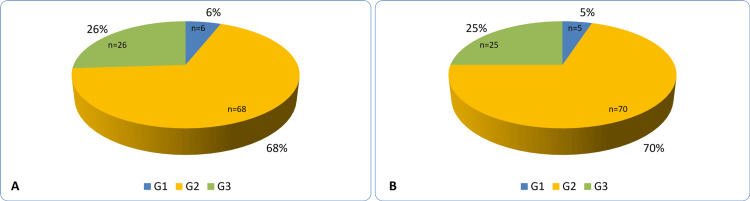
Tumor grade distribution of (A) screen‑detected breast cancers and (B) symptomatic breast cancers G1: Grade 1; G2: Grade 2; G3: Grade 3.

Pathological grade distributions were similar between the groups (G1: 5% vs. 6%; G2: 70% vs. 68%; G3: 25% vs. 26% in SBC and SDBC, respectively; Figure 2).

**Figure 3:**
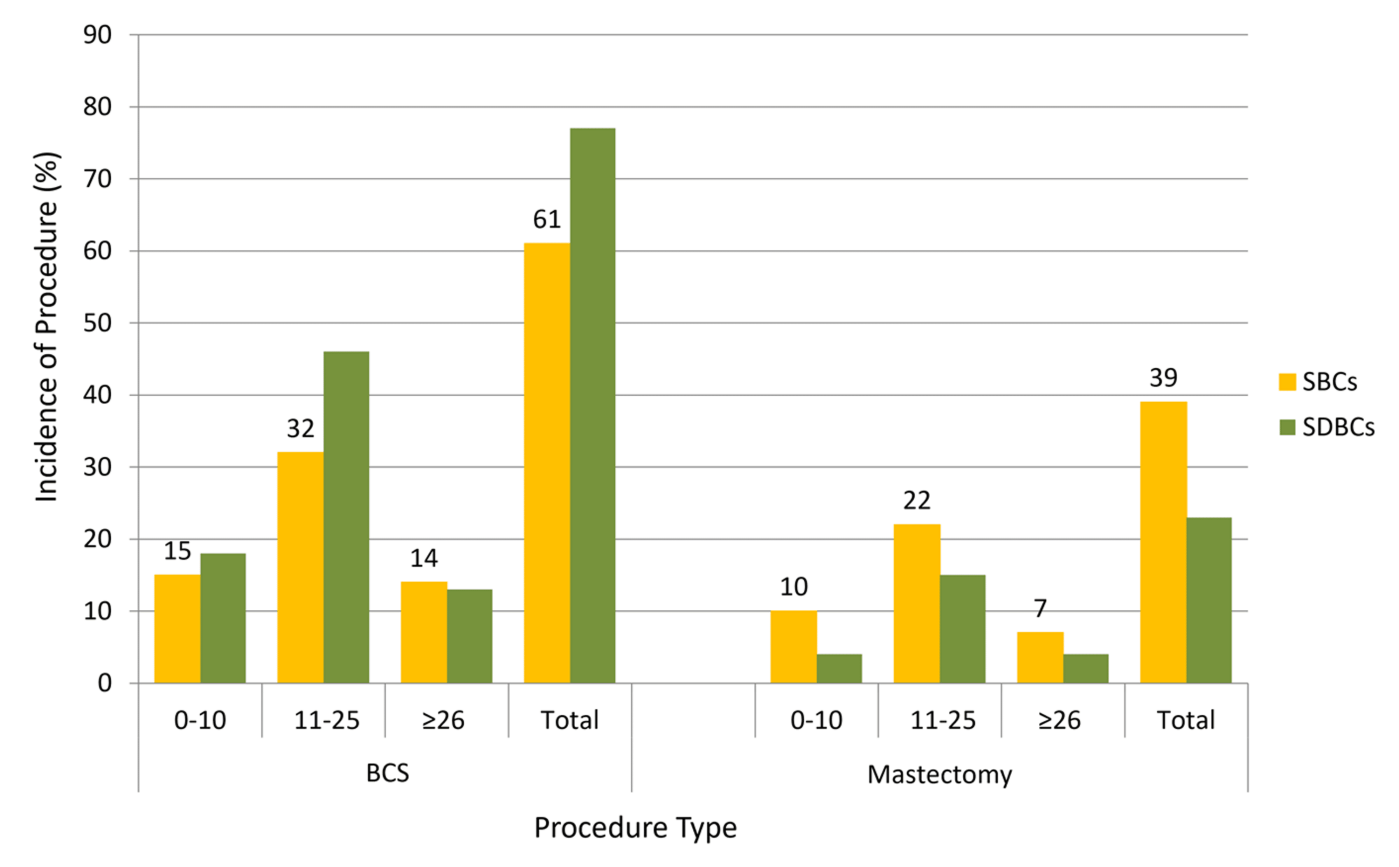
Surgical procedures in the diagnostic categories by ODX‑RS subgroup BCS: breast conservation surgery; ODX-RS: Oncotype DX Recurrence score.

However, SDBCs showed a higher frequency of HER2 immunohistochemistry 1+ (low HER2 expression) scores (54% vs 38%; P=0.014). SDBCs were also more likely to have borderline HER2 expression (32% vs 20%; P=0.05) and invasive lobular carcinoma (25% vs 19%; P=0.3) compared with SBCs. BCS was performed in 77% of SDBC cases and 61% of SBC cases, while mastectomy was more frequent in the SBC group (39% vs 23%; P=0.01; Figure 3).

**Figure 4:**
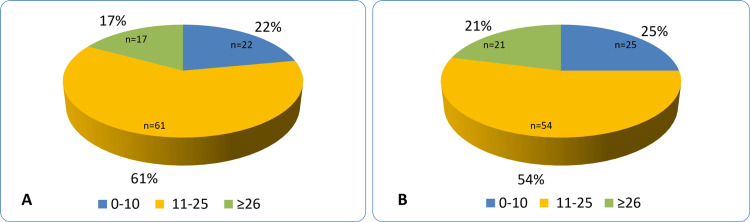
Oncotype DX Recurrence score distribution: (A) screen detected breast cancers, (B) symptomatic breast cancers ODX-RS: Oncotype DX Recurrence score.

ODX-RS ≥26 was observed slightly more often in the SBC group than in the SDBC group (21% vs 17%; P=0.47) (Figure 4).

**Figure 5:**
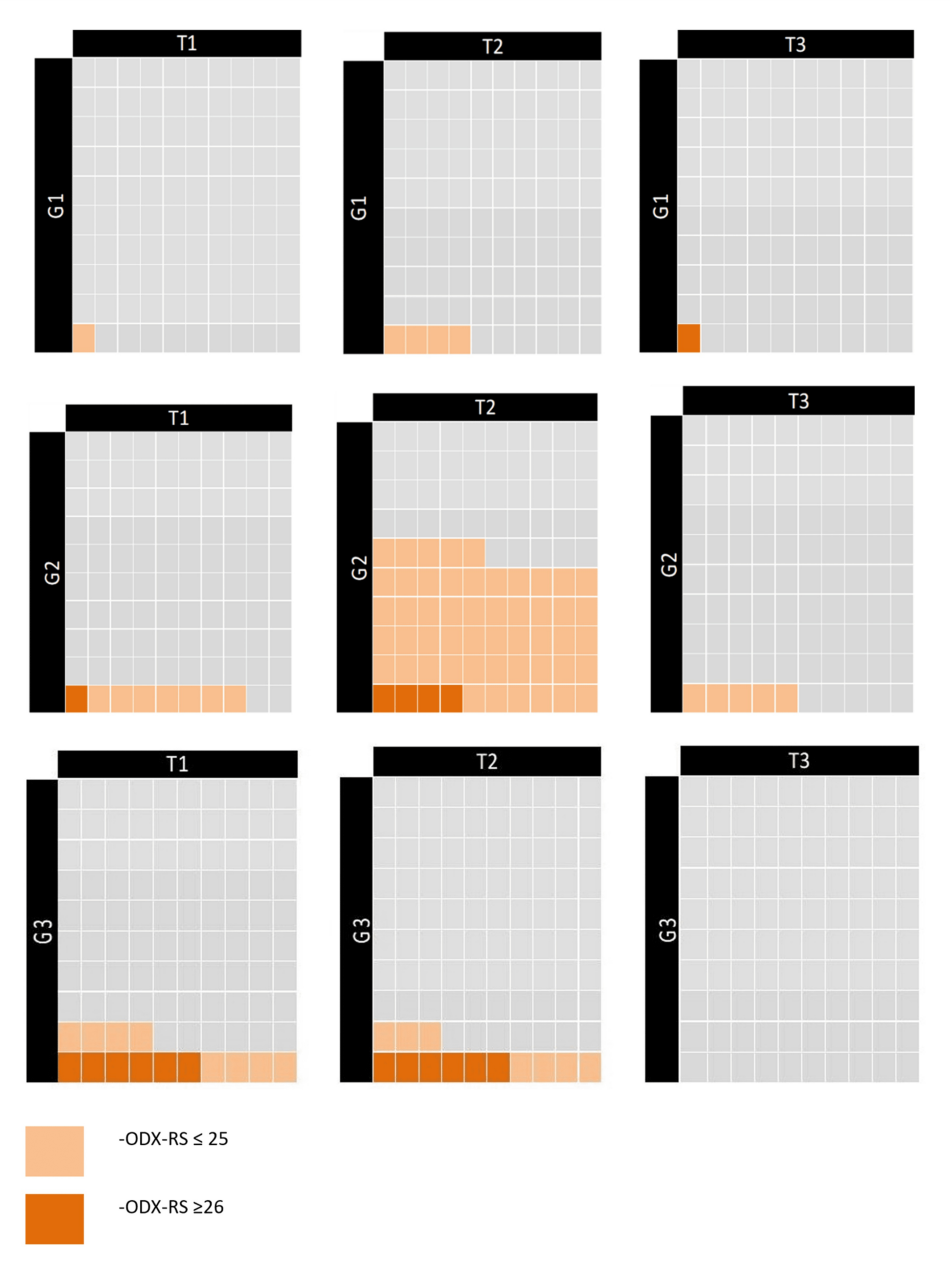
ODX‑RS by tumor size and grade in screen‑detected breast cancers ODX-RS: Oncotype DX Recurrence score. Created by the author Abdalla Saad Abdalla Al-Zawi.

**Figure 6:**
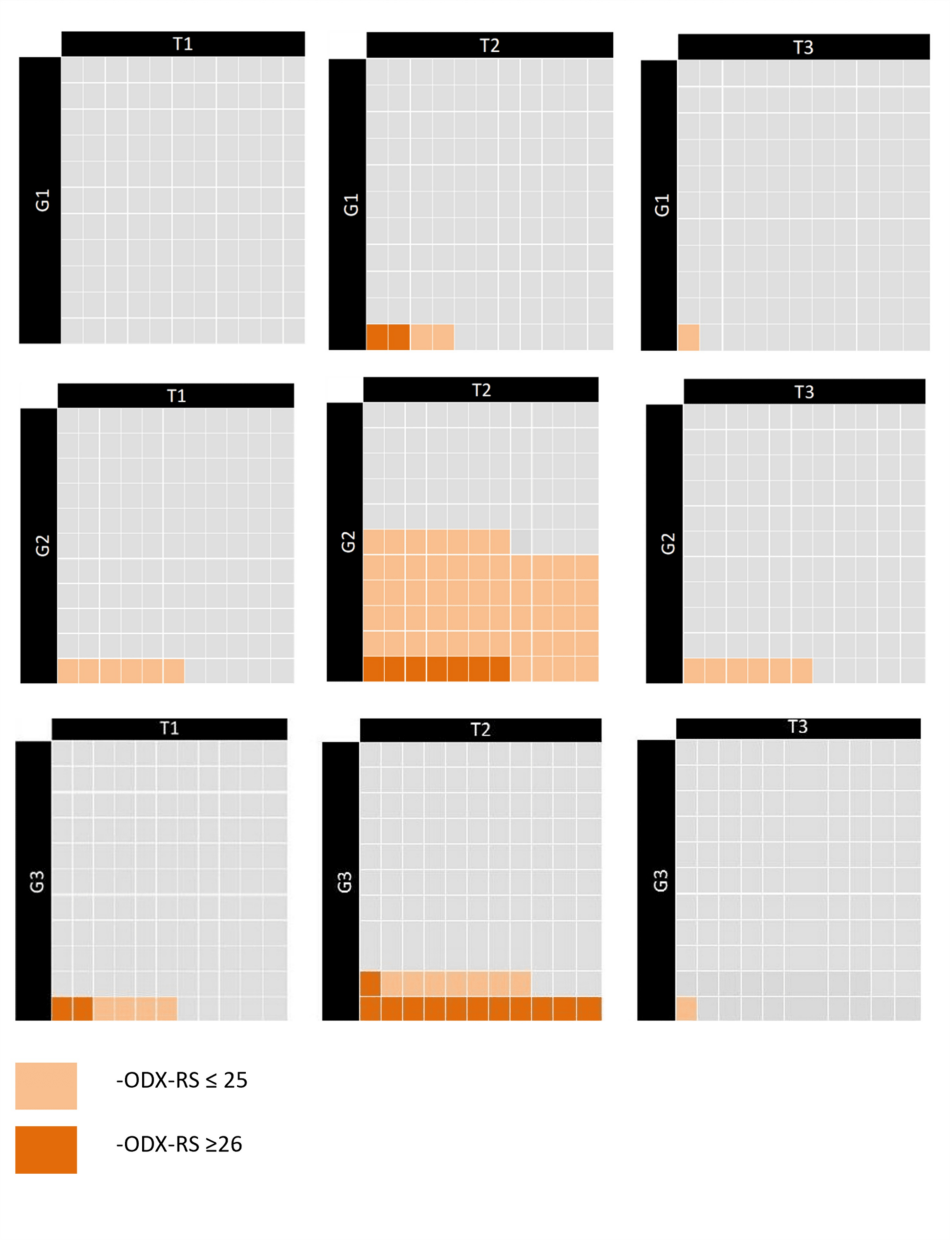
ODX‑RS by tumor size and grade in symptomatic breast cancers ODX‑RS: Oncotype DX Recurrence score. Created by the author Abdalla Saad Abdalla Al-Zawi.

In patients with ODX-RS ≥26, BCS was performed in 14% of SDBC cases and 13% of SBC cases (P=0.05; Figure 5). Subgroup analysis of ODX-RS based on tumor size and histologic grade showed similar distributions. The largest subgroup (grade 2, T2 tumors with ODX-RS ≤25) accounted for 55% of SBCs and 51% of SDBCs (P=0.33; Figures 5, 6).

For Grade 3, T1 tumors with ODX-RS ≤25, the rates were 8% in SBCs and 4% in SDBCs (P=0.69). Grade 3, T2 tumors with ODX-RS ≥26 were reported in 11% of SDBC cases and 6% of SBC cases (P=0.29).

**Figure 7:**
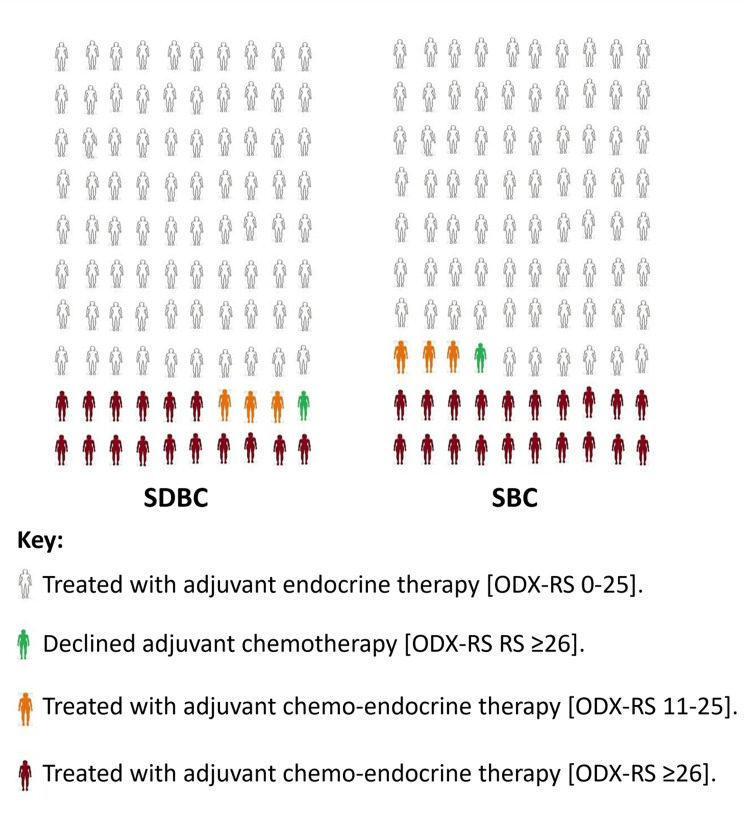
Adjuvant chemotherapy recommendations based on ODX‑RS ODX‑RS: Oncotype DX Recurrence score; SDBC: screen‑detected breast cancer; SBC: symptomatic breast cancer. Created by the author Abdalla Saad Abdalla Al-Zawi.

SDBCs showed a slightly higher likelihood of indicating adjuvant chemotherapy (23% vs 19%; P=0.49), possibly reflecting the marginally higher rate of ODX-RS ≥26. In contrast, SBCs had a slightly higher likelihood of receiving adjuvant endocrine therapy (81% vs 77%; P=0.49; Figure 7).

## Discussion

Since the 1990s, declines in breast cancer-related mortality have been observed in several countries. These reductions have been attributed to multiple factors, including the implementation of national breast cancer screening programs, increased public awareness, and earlier detection of palpable tumors through education on regular breast self-examination [3,14,15]. Other contributing factors include reductions in breast cancer risk due to improved adjuvant systemic therapies with fewer side effects, more appropriate recommendations for systemic therapy, and the adoption of personalized management strategies [16-18].

White et al. analyzed data from 30,560 breast cancer cases diagnosed in Ireland between 2009 and 2018. Of these, 40% were within the target age for screening, and 25% were screen-detected [19]. Similarly, a 2024 report by Barclay et al., based on 5,848,436 eligible women and 5,539,681 men aged 18 years or older using data from the UK Clinical Practice Research Datalink GOLD and Aurum databases, found that one-year breast cancer survival increased by 2.08 percentage points and five-year survival increased by 5.39 percentage points between 2000-2004 and 2015-2019, particularly among women aged 50-70 years. The authors concluded that improvements in overall survival were likely due to the success of screening programs, early detection, and enhancements in treatment pathways [20].

Emerging evidence suggests that artificial intelligence may improve breast cancer detection rates in screening mammography. It may also help reduce the image-reading workload for radiologists [21].

SDBCs are typically smaller and exhibit more indolent biological behavior than SBCs. Clinicians often ask whether these cancers, if left undiagnosed during the asymptomatic or preclinical phase, would merely increase in size or also become more aggressive [7]. Tumor size may serve as a proxy for disease duration and, in screen-detected cancers, reflects the length of the preclinical phase [22]. The average risk of axillary lymph node involvement is lower in asymptomatic breast cancers; however, when nodal metastases are present, they often indicate underlying biological aggressiveness and prolonged disease duration [23].

Even within screen-detected cancers, differences exist. Interval breast cancers, diagnosed between screening rounds, tend to have more aggressive phenotypes than those detected during routine screenings. They are more likely to be of higher pathological grade, lobular subtype, and triple-negative [16]. Interval cancers also display greater genomic instability, including higher mutation rates, structural chromosomal abnormalities, elevated Ki-67 expression, altered hormone receptor activity, defective homologous recombination, and TP53 mutations [7,16].

Crispo et al. investigated the molecular profiles of screen-detected versus symptomatic breast cancers in 448 patients with operable disease. Screen-detected tumors had less aggressive clinicopathological features and better survival outcomes. Notably, patients with luminal A subtype SDBCs had significantly longer disease-free survival and disease-specific survival [24].

Consistent with previous findings, SDBCs in our study had higher rates of BCS. Fancellu et al. reported that 89.1% of SDBCs underwent BCS compared with 59.1% of SBCs (P<.01) in a cohort of 733 patients [14]. Our results showed BCS was performed in 77% of SDBCs versus 61% of SBCs (OR=0.47, P=0.01) (Figure 4). Starikov et al. reported similar findings in a cohort of 1,026 patients, with SDBCs being smaller (median size 8 mm vs 15 mm, P<.001), less invasive (80.8% vs 94.3%), and more frequently treated with BCS (74.8% vs 59.9%, P<.001). Our findings are consistent: BCS was performed in 77% of SDBCs and 61% of SBCs (OR=0.47, P=0.01). In terms of adjuvant chemotherapy, Starikov et al. found it was used in 51.3% of SBCs versus 20% of SDBCs (P<.001). In our study, 23% of SBCs and 19% of SDBCs received chemotherapy (OR=1.27, P=0.49) [5].

The integration of genomic assays into early breast cancer management has allowed clinicians to better estimate recurrence risk and predict the benefit of adjuvant systemic therapy. This supports tailored treatment decisions, enabling physicians to identify patients who may benefit from chemotherapy while sparing others from unnecessary treatment and associated toxicities [25].

Among patients with early-stage breast cancer, those with a high-risk recurrence score on the 21-gene ODX-RS assay benefit from adjuvant chemotherapy in addition to endocrine therapy, whereas those with low-risk scores typically do not [11-13]. While previously no data were published comparing ODX-RS differences between SDBCs and SBCs, our study suggests that such differences may exist and merit further exploration.

In subgroup analyses stratified by tumor size and histological grade, most subsets showed similar ODX-RS distributions between SDBCs and SBCs. The largest subgroup, Grade 2, T2 tumors with ODX-RS ≤25, comprised 55% and 51% of SBCs and SDBCs, respectively. Notably, Grade 3, T2 tumors with ODX-RS ≥26 were more frequent among SBCs than SDBCs. Despite this, both groups had nearly identical BCS rates among patients with ODX-RS ≥26: 13% in SDBCs and 14% in SBCs (OR=0.61, P=0.05). Additionally, SBCs had a slightly higher rate of chemotherapy indication (23% vs 19%; OR=1.27, P=0.49), which aligns with previous findings on differential chemotherapy use between symptomatic and screen-detected cancers [5,26].

Limitations

This study has several limitations that warrant consideration. First, its retrospective design is subject to selection and information biases because data were obtained from existing clinical records, which may be incomplete or inconsistently documented. Second, the cohort was modest (100 SDBCs and 100 SBCs from a single regional hospital network), reducing statistical power and limiting generalizability to broader or more diverse populations.

Third, the analysis did not include long-term endpoints such as disease-free survival or overall survival, so we could not correlate ODX-RS categories with actual recurrence or mortality. Fourth, potential confounders, including comorbidities, socioeconomic factors, screening intervals, and variability in surgical or systemic treatment, were not systematically recorded and could influence both presentation mode and treatment decisions. Finally, the study period spanned 2017-2025, during which screening guidelines and adjuvant therapy protocols evolved; temporal changes may have affected management patterns and ODX-RS ordering practices. Prospective, multicenter studies with larger, more diverse cohorts and standardized follow-up are needed to validate these observations and clarify the impact of presentation mode on clinical outcomes.

## Conclusions

This study evaluated whether the clinical differences between breast cancers detected through screening mammography (SDBCs) and those diagnosed after symptom onset (SBCs) are reflected in the ODX-RS. Our study did not identify a clinically meaningful difference in genomic risk between cancers detected through routine screening and those diagnosed after symptom onset. Although screen-detected tumors tended to be smaller and were more often managed with breast-conserving surgery, their Oncotype DX risk profiles and the resulting systemic treatment recommendations were largely similar to symptomatic cases. Presentation status should thus be viewed as a complementary descriptor, not a surrogate for genomic risk, when tailoring adjuvant therapy. Larger prospective studies with long-term outcomes are warranted to confirm these observations and to explore whether integrating presentation mode with genomic assays can further refine personalized care.
